# Iatrogenic ventricular septal defect after transcatheter aortic valve implantation: a rare complication

**DOI:** 10.21542/gcsp.2024.55

**Published:** 2024-12-31

**Authors:** Muhammad Salman Sabri, Hussam Al Hennawi, Shayan Qadir, Lucy Checchio, Chaitra Janga, Hamza Muhammadzai, Donald Haas

**Affiliations:** 1 Department of Internal Medicine, Jefferson Abington Hospital, PA, USA; 2 Department of Cardiology, Thomas Jefferson University Hospital, PA, USA; 3 Department of Comprehensive Heart Failure, Jefferson-Abington Hospital, PA, USA

## Abstract

Iatrogenic ventricular septal defect (VSD) is a rare complication following transcatheter aortic valve implantation (TAVI). An 83-year-old male underwent TAVI for severe aortic stenosis (AS) and was diagnosed with a peri-membranous VSD on echocardiography, which was not evident on pre-procedural imaging. This case highlights the risk factors, symptoms, diagnosis, and management of iatrogenic VSD following TAVI.

## Introduction

In adults, ventricular septal defect (VSD) is the second most common congenital abnormality after bicuspid aortic valve^1^. One of the rare causes of VSD is iatrogenic after transcatheter aortic valve implantation (TAVI). TAVI can rarely cause membranous or muscular VSD^2^. We present a unique case of membranous VSD that was accidentally diagnosed on transthoracic echocardiography two weeks after TAVI and subsequent dual-chamber pacemaker implantation due to iatrogenic complete heart block. We will also discuss the emphasis and timeline of post-TAVI echocardiography to identify any asymptomatic procedural complications and timely intervention.

## Case

An 83-year-old male with a significant medical history (as mentioned below) presented to the hospital with complaints of melena, coffee-ground emesis, and shortness of breath. His medical hisotry included hypertension, hyperlipidemia, ischemic cardiomyopathy, atrial fibrillation managed with apixaban, coronary artery disease requiring coronary artery bypass grafting (CABG), and severe aortic stenosis (AS) – for which he underwent TAVI with an Edwards Sapien valve two weeks prior to admission – and prostate cancer. The TAVI procedure was complicated by complete heart block, necessitating the placement of a dual-chamber pacemaker.

**Figure 1.**
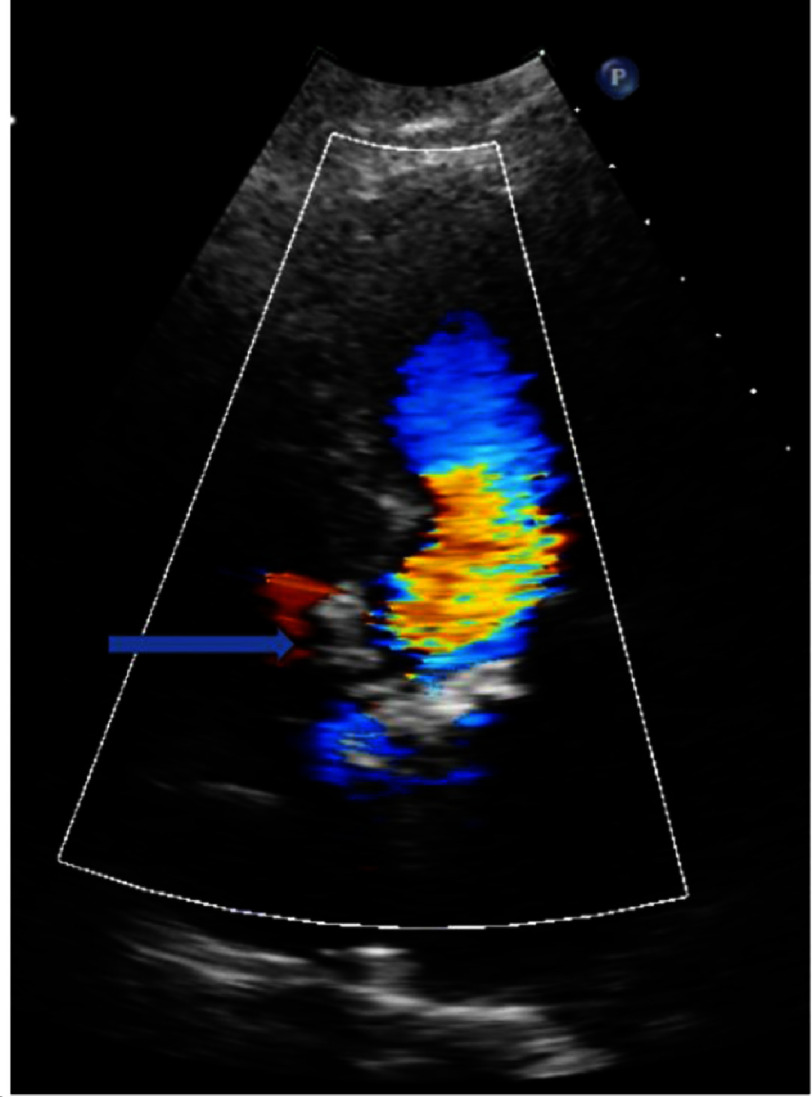
Transthoracic echocardiography demonstrating membranous ventricular septal defect.

**Figure 2.**
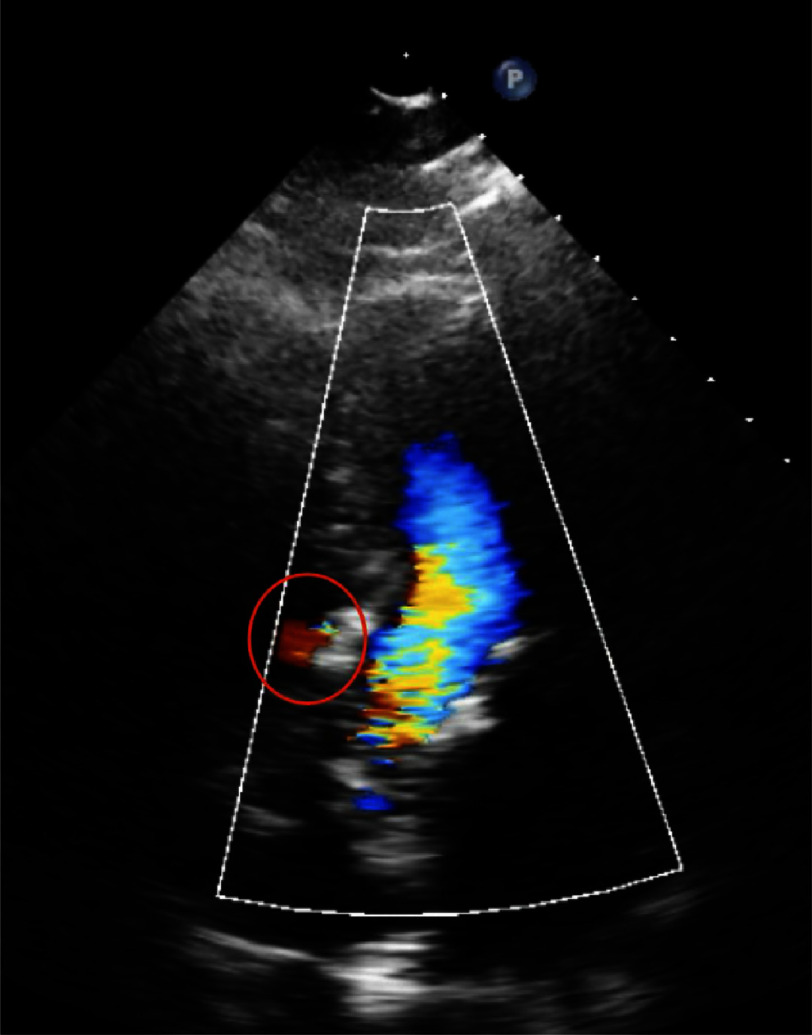
5-chamber view of transthoracic echocardiogram demonstrating membranous ventricular septal defect.

**Figure 3.**
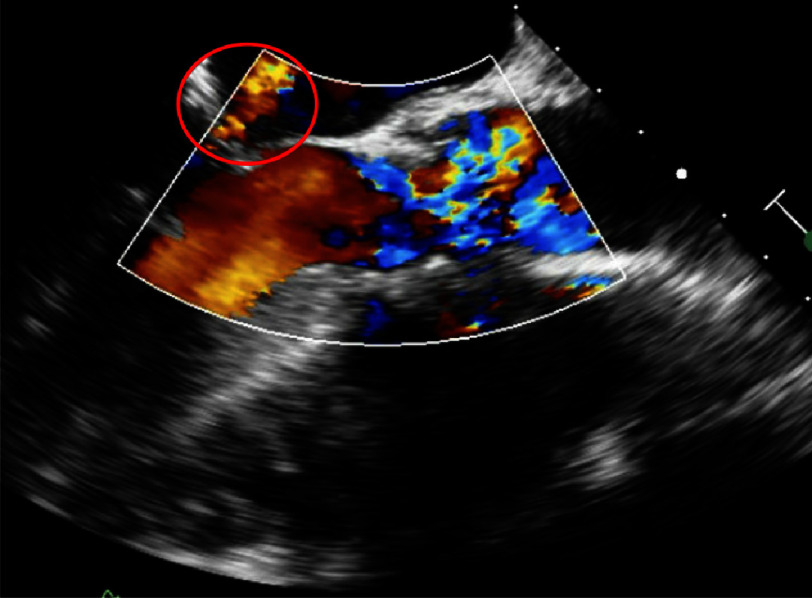
Transesophageal echocardiography demonstrating a small peri membranous ventricular septal defect with trivial left to right shunting by color flow Doppler.

He underwent a transthoracic echocardiogram (TTE) prior to TAVI, which revealed severe aortic stenosis with a mean gradient of 73 mmHg, significant calcification of the aortic annulus and leaflets, with no evidence of a VSD. His physical examination was significant for tachycardia, systolic murmur, and bilateral lower extremity edema. The blood tests showed a hemoglobin level of 4.9 g/dL, down from a baseline of 9.8 g/dL, elevated white blood cell count of 29,700 cells per microliter, elevated potassium at 5.1 mmol/L, and creatinine at 1.35 mg/dL, mildly elevated from his baseline of 1.05 mg/dL. He also underwent a TTE that indicated the presence of a membranous VSD as shown in Figure 1 and Figure 2. Transesophageal echocardiography (TEE) demonstrated a very small peri membranous VSD with trivial left to right shunting by color flow Doppler as depicted in Figure 3.

To assess the VSD’s extent and determine the need for intervention, the patient underwent right heart catheterization to evaluate right heart hemodynamics. The study revealed mild pulmonary hypertension and a small left-to-right shunt at the level of the high right ventricle (RV) with oximetry saturation of 72% in high RV and 76% in the RV outflow tract and pulmonary-systemic shunt ratio (QP: QS) of 1.46. His right atrial (RA) pressure was 3 mm Hg, and pulmonary capillary wedge pressure (PCWP) was 7 mm Hg. His cardiac output and cardiac index were 5.48 L/min and 3.1 L/min/m^2^, respectively. The patient continued to improve from a cardiac standpoint.

However, the patient had a complicated course including respiratory arrest, requiring prolonged intubation and tracheostomy, due to ventilatory dependent respiratory failure. He developed septic shock due to multi-drug resistant Klebsiella and Corynebacterium. Furthermore, he had hematuria, due to radiation cystitis from his treatment of previous prostate cancer, for which he required continuous bladder irrigation and clot evacuation. He required bilateral nephrostomy tubes due to extravasation of urine from left lateral wall and also developed peribladder density collection concerning for bladder rupture. He had persistent bacteremia and underwent multiple echocardiograms including a transesophageal echocardiogram. His last echocardiogram showed 2 vegetations, one on the mitral valve and the other on the prosthetic aortic valve. However, due to patient frailty, the patient was not deemed a surgical candidate for any intervention. He ultimately developed end stage renal disease requiring continuous renal replacement therapy (CRRT), which could not be tolerated due to continued hypotension. He developed lactic acidosis from septic shock, culminating in cardiac arrest and eventual demise.

**Table:** 

Timing	Events
2 weeks prior to index admission	TAVR procedure with Edward Sapien valve
Day 1	Admitted due to acute blood loss anemia. Hemoglobin of 4.9 g/dL. TTE done due to shortness of breath. Found to have membranous VSD.
Day 4	Esophagogastroduodenoscopy performed. Clean based ulcer found which was likely source of bleed.
Day 6	Right heart catheterization. Qp:Qs of 1.46. Oxygen saturation 72% in RV and 76% in RVOT.
Day 9	Respiratory arrest, resulting in PEA arrest. Echocardiogram after resuscitation showed preserved LV function, and VSD.
Day 25	Prolonged ventilator dependent respiratory failure, requiring tracheostomy and percutaneous endoscopic gastrostomy tube.
Day 30	Septic shock due to MDR Klebsiella and Corynebacterium.
Day 44	Hematuria requiring CBI and clot evacuation.
Day 50	Bilateral nephrostomy tubes due to bladder rupture.
Day 52–74	Persistent bacteremia requiring multiple echocardiograms. Vegetation seen on the last echocardiogram on mitral valve and prosthetic aortic valve. Patient too frail for surgery.
Day 80	End stage renal disease requiring HD.
Day 81	Hypotension leading to medical ICU upgrade.
Day 82	Lactic acidosis despite vasopressor support and continuous replacement therapy. Cardiac arrest and ultimate demise.

## Discussion

Iatrogenic ventricular septal defect is a recognized but uncommon complication following surgical valve placement^3^. This complication typically occurs after mitral or tricuspid valve replacement due to increased vulnerability to tearing forces across the right fibrotic trigone.

One of the rare causes of iatrogenic VSD is TAVI. Patients with iatrogenic VSD after TAVI can be asymptomatic or experience new shortness of breath, worsening of dyspnea, or chest pain^2^. A systemic analysis that included 20 patients^4^ with iatrogenic VSD after TAVI demonstrates that clinical presentation varied from asymptomatic to heart failure symptoms. A retrospective study^5^ analyzed 400 patients who underwent TAVI, revealing that 6 patients experienced VSD as a post-procedural complication. Among them, 4 patients developed membranous VSD, and 2 patients had muscular VSD, all of whom were asymptomatic^5^. TTE with color Doppler study is the best initial diagnostic test for VSD^1^. Transesophageal echocardiography (TEE) can be considered if there is a high suspicion of VSD with a negative TTE^1^.

Diagnosis of post-TAVI VSD can vary from immediate identification to a year^4^. Our patient did not undergo a post-TAVR TTE, but a TTE conducted after 2 weeks revealed a well-positioned Sapien bioprosthetic valve and a small peri-membranous VSD with minimal left-to-right shunting observed on color Doppler flow. The pressure exerted by the valve on the ventricular septum may contribute to VSD formation^5^. During TAVI, preventing paravalvular regurgitation involves using transcatheter heart valves larger than the annular size^7^. Valvular oversizing can lead to aortic annular rupture and subsequent VSD formation. This complication has been documented with Sapien valves but not with Core valves^5^. Low implantation of the self-expanding Core valve also plays a role in VSD, and it can present as delayed iatrogenic VSD^5^. Post-TAVR VSD may also arise from calcium deposits at the aortic root annulus, which can displace the bioprosthetic valve towards the membranous septum or be pushed by the valve itself, potentially leading to VSD^5^. In our patient, the likely cause of the iatrogenic VSD was valvular oversizing with the Sapien valve, in combination with significant aortic annular calcification. CT and magnetic resonance imaging (MRI) may be considered for further evaluation of VSD anatomy^6^. Cardiac catheterization is seldom necessary for diagnosing VSD but can be utilized to calculate the Qp: Qs ratio when evaluating the need for VSD closure.

The management of post-TAVI VSD ranges from conservative management to surgical management. It depends on the patient’s symptoms and the size of the VSD^2^. Asymptomatic and small VSDs can be managed conservatively with regular monitoring^2^. Symptomatic and hemodynamically significant VSD should be ideally closed^1,2,5^. VSD management can be surgical or percutaneous closure, which depends on comorbidities, the size of VSD, and the surgical risk of the patient. In patients with high surgical risk, percutaneous closure is preferred over surgical closure^2,4^.

## Conclusion

Iatrogenic VSD is a rare complication of TAVI. The diagnosis may be delayed if symptoms are absent or if a post-TAVI TTE appears normal. Patients at risk for post-TAVI VSD, such as those with aortic annular calcification, use of an oversized valve, or low implantation of a self-expandable valve, should undergo careful screening. The management of VSD depends on patient symptoms, hemodynamics, and size of the VSD.
